# Flow vortex quantification in the left atrium

**DOI:** 10.1186/1532-429X-14-S1-W23

**Published:** 2012-02-01

**Authors:** Prasanta Pal, Ziheng Zhang, Ben A Lin, Donald Dione, Albert J Sinusas, Smita Sampath

**Affiliations:** 1 Diagnostic Radiology, Yale University School of Medicine, New Haven, CT, USA; 2 Cardiology, Yale University, New Haven, CT, USA

## Summary

We have developed a novel high temporal resolution magnetic resonance (MR) imaging and image analysis method to quantify vorticity patterns in the left atrium (LA). Our data demonstrates the formation and evolution of atrial vortices during ventricular systole and ventricular diastasis period.

## Background

Recent studies indicate that up to 25% of patients with heart failure (HF) develop atrial fibrillation (AF). Current imaging markers of LA dilatation and LA fibrosis are detectable only at the late stage of atrial remodeling when irreversible structural changes have occurred. Functional imaging markers of LA remodeling are severely lacking. Abnormalities in LA flow patterns may provide a non-invasive quantitative approach to study LA function and may be detectable early.

## Methods

We have developed a novel high temporal resolution phase contrast MR imaging pulse sequence. This sequence was used to acquire LA velocity data in two orthogonal directions on a 4-chamber slice with imaging parameters: imaging matrix: 192×192, resolution: 1.5mm×1.5mm, slice thickness: 8mm, views per cardiac phase: 3, Venc: 120-150 cm/s, temporal resolution: 15-16ms. The velocity data was filtered using Gaussian filters with appropriate averaging radius. The filtered data was used to generate the two-dimensional velocity flow field. Vorticity at every point was calculated from the curl of the velocity field using the following equation.

v=(1/N^2) ∑∑((V_y(i+m,j+n)-V_y(i-m,j-n))/(2mΔx)-(V_x(i+m,j+n)-V_x(i-m,j-n))/(2nΔy))

A finite element differentiation technique was employed to compute velocity field differentiations using sampled data within a defined interrogation window (of size N). The numerical calculations were carried out using these sampled vectors along contours defined around the given point in the flow field. Positive vorticity values signify counter-clockwise rotation, while negative values signify clockwise rotation.

## Results

**Figure 1:**
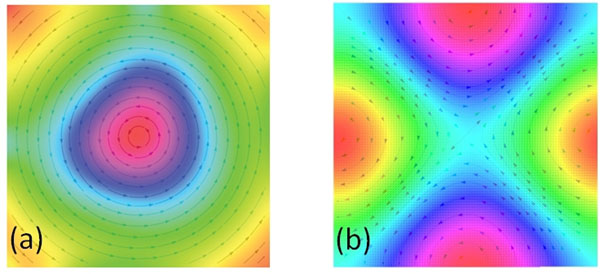
Computed vorticity patterns from simulated flow fields depict (a) single counterclockwise vortex, and (b) four vortices-two clockwise and two anticlockwise vortices.

**Figure 2:**
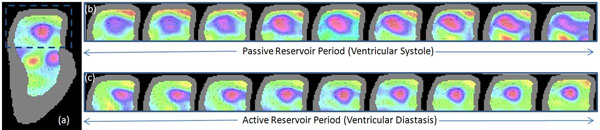
(a) Vorticity color map in a normal volunteer during one frame in ventricular diastasis. Vortices are seen in the atrium and the ventricle. Temporal patterns of vorticity maps during (b) ventricular systole and (c) ventricular diastasis in the left atrium depict formation and decay of the atrial vortices.

Simulated velocity fields (streamlines) and their superimposed vorticity color maps obtained using our image analysis methods are shown in Fig 1a and 1b. A single vortex is seen in Fig. 1a, while four separate vortices (two clockwise and two anticlockwise) are seen in Fig. 1b. Streamlines with overlay of vorticity color maps in a normal volunteer dataset is also illustrated in Fig. 2. An atrial vortex is observed during ventricular systole and ventricular diastasis. The vorticity maps clearly depict the formation and evolution of these atrial vortices.

## Conclusions

Vortices are formed in the left atrium during early systole and during the diastasis period of the cardiac cycle. These characteristic vortex patterns have been quantified from the 2D flow fields through finite difference curl operator.

